# Subclinical Highly Pathogenic Avian Influenza Virus Infection among Vaccinated Chickens, China

**DOI:** 10.3201/eid2012.140733

**Published:** 2014-12

**Authors:** Qing-Xia Ma, Wen-Ming Jiang, Shuo Liu, Su-Chun Wang, Qing-Ye Zhuang, Guang-Yu Hou, Xiang-Ming Liu, Zheng-Hong Sui, Ji-Ming Chen

**Affiliations:** College of Marine Life Sciences, Ocean University of China, Qingdao, China (Q.-X. Ma, Z.-H Sui);; China Animal Health and Epidemiology Center, Qingdao (W.-M. Jiang, S. Liu, S.-C. Wang, Q.-Y. Zhuang, G.-Y. Hou, X.-M. Liu, J.-M. Chen);; Qingdao Center for Control of Animal Infectious Diseases, Qingdao (Q.-X. Ma)

**Keywords:** Influenza, type A, subtype H5, H5N2, avian influenza virus, pathogenicity, epidemiology, vaccine-escape, variant, zoonosis, poultry, viruses, subclinical, silent infection, HPAI, highly pathogenic avian influenza, vaccine, China

## Abstract

Subclinical infection of vaccinated chickens with a highly pathogenic avian influenza A(H5N2) virus was identified through routine surveillance in China. Investigation suggested that the virus has evolved into multiple genotypes. To better control transmission of the virus, we recommend a strengthened program of education, biosecurity, rapid diagnostics, surveillance, and elimination of infected poultry.

Highly pathogenic avian influenza (HPAI) type A viruses of the subtype H5 have been circulating among poultry in several countries of Asia and Africa for many years. HPAI viruses of this subtype have also caused hundreds of infections among humans and presented a substantial threat to public health (*1*,*2*). China is one of the countries deeply affected by zoonotic viruses of this subtype (*3*). The government of China decided during 2005 to use a comprehensive strategy to control this zoonosis involving mass vaccination of poultry and strict culling of infected flocks (*2*). Although this strategy has played a successful role in guaranteeing safety of food supplies, maintaining the poultry system, and minimizing human infections, governmental agencies in China are considering how to exit from mass vaccination programs, mainly because of the tremendous cost of this intervention. We report here an investigation of recent subclinical circulation of the HPAI virus among egg-laying chickens on a farm in China, to provide novel data and views on the evolution of the virus and improvement of HPAI control in China.

## The Study

During routine surveillance in January 2014, we collected 30 swab samples from chickens on an egg-laying chicken farm populated by 12,000 320-day-old and 6,000 150-day-old chickens without any clinical signs, in Qingdao, Shandong Province, China. Of those, 5 were positive by real-time reverse transcription PCR for detection of the hemagglutinin (HA) gene of H5 HPAI viruses. The flock was culled immediately after the diagnosis, and an H5N2 subtype HPAI virus was isolated by inoculating specific-pathogen-free embryonated eggs with the collected samples. The entire viral genome of the virus, A/chicken/Qingdao/1/2014(H5N2), abbreviated as H5N2qd14, was sequenced and analyzed as described (*4*). The sequences were deposited in GenBank under accession nos. KJ683877–KJ683884. We used the full-length sequences for each gene in phylogenetic analyses.

The HA protein of the H5N2qd14 virus has multiple basic amino acid residues (PQIEGRRRKR↓GLF) at the cleavage site, categorizing it as an HPAI virus. Its intravenous pathogenicity index is 2.84, determined by intravenously inoculating 10 chickens, which were 6 weeks old and specific pathogen–free, with 0.1 mL of a 1:10 dilution of the H5N2qd14 allantoic fluid. Phylogenetic analysis of the HA gene suggested that H5N2qd14 is a variant of clade 7.2 (Figure); this variant has become predominant in clade 7.2 in China since 2011 (*5*). Re-4, the vaccine strain which corresponds to this clade, was generated in 2006 through reverse genetics: the HA gene was derived from the virus A/chicken/Shanxi/2/2006 (*2*). As compared with the vaccine strain, 47 aa mutations occurred in the HA protein of H5N2qd14, of which 9 (I124V, H126R, G154E, K155N, L162V, I166T, T182A, V189I, D198N) were located on the antigenic epitopes of the viral protein, suggesting that H5N2qd14 is likely distinct in antigenicity from the vaccine strain (*6*,*7*). This conclusion is supported by our epidemiologic investigation, which revealed that the affected flock had been vaccinated 3 times with the vaccine strain Re-4. Moreover, we collected 30 serum samples from the flock simultaneously with the swab samples, and analyzed them using the hemagglutination inhibition (HI) assay. The geometric mean and standard deviation of the HI titers of these 30 serum samples were 250.51 ± 2.93 against the vaccine strain Re-4 and 10.83 ± 2.79 against H5N2qd14, showing a 23-fold difference. Additionally, the geometric mean and standard deviation of the HI titers of 20 standard serum specimens specifically against the vaccine strain Re-4, which we prepared in-house using specific pathogen–free chickens, were 388.02 ± 1.92 against the vaccine strain Re-4 and only 4.92 ± 1.48 against H5N2qd14, with a 78-fold difference.

**Figure:**
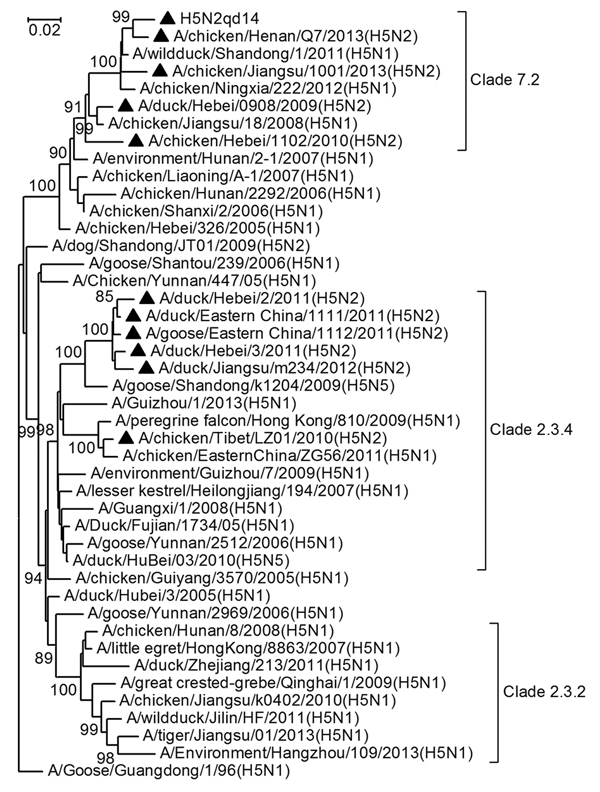
The phylogenetic relationships among some subtype H5 highly pathogenic influenza viruses based on their heamagluttinin sequences. The H5N2 subtype viruses identified in China in recent years are marked with black triangles. Bootstrap values are given at relevant nodes.

Phylogenetic analysis of the genomic sequences further suggested that the 2 genomic segments of H5N2qd14 coding the HA and matrix protein genes were likely from recent H5N1 HPAI viruses in clade 7.2, and the remaining 6 genomic segments of H5N2qd14 likely reassorted from subtype H9N2 avian influenza virus (Figure, Technical Appendix Figures 1–7). Moreover, analysis of the viral genomic sequences available in the Global Initiative on Sharing Avian Influenza Data (http://platform.gisaid.org/epi3/frontend#216031) suggested that H5N2 HPAI viruses have evolved into ≥2 HA clades (clades 7.2 and 2.3.4) and 6 genotypes in China in recent years (Table, Technical Appendix Figures 1–7). The genotype corresponding to H5N2qd14 has been identified in 2 other provinces, Henan and Jiangsu, in China during 2013 (Table). The H5N2 HPAI viruses in clade 2.3.4 are also likely vaccine-escape variants as suggested by the similar phylogenetic and antigenic analysis given above (data not shown).

**Table:** Six genotypes of highly pathogenic avian influenza type A identified during 2009–2014 calculated through phylogenetic analysis, China

Genotype	Representative virus	Clade	Lineage of gene*							
			PB2	PB1	PA	HA	NP	NA	M	NS
1	H5N2qd14	7.2	A	A	A	B	A	A	B	A
	A/chicken/Jiangsu/1001/2013(H5N2)	7.2	A	A	A	B	A	A	B	A
	A/chicken/Henan/Q7/2013(H5N2)	7.2	A	A	A	B	A	A	B	A
2	A/chicken/Hebei/1102/2010(H5N2)	7.2	A	A	A	B	A	A	A	A
3	A/duck/Hebei/0908/2009(H5N2)	7.2	B	B	B	B	B	B	C	B
4	A/duck/Hebei/2/2011(H5N2)	2.3.4	C	C	C	C	C	C	D	C
5	A/duck/Jiangsu/m234/2012(H5N2)	2.3.4	C	B	C	C	C	B	D	C
6	A/chicken/Tibet/LZ01/2010(H5N2)	2.3.4	D	A	A	D	A	A	A	D
*Lineages of each gene are partially shown in online Technical Appendix Figures 1–7 (wwwnc.cdc.gov/eid/article/20/12/14-0733-techapp1.pdf). Lineage A of each gene is likely from H9N2 subtype avian influenza virusess. Any 2 viruses with >1 gene of different lineages belong to different genotypes. PB, polymerase basic protein; PA, polymerase acidic protein; HA, hemagglutinin; NP, nucleocapsid protein; NA, neuraminidase; M, matrix protein; NS, nonstructural protein.										

No mutations in the viral HA protein that confer binding to human-like receptors were identified, suggesting that this virus likely cannot bind efficiently to human-like receptors (*8*). No mutations in the viral polymerase basic 2 protein, which are associated with high pathogenicity of the virus in mammals, were identified (*9*). No mutations in the viral neuraminidase protein (NA), which confers resistance to oseltamivir, were identified (*10*). However, a mutation of S31N occurred in the viral matrix 2 protein, which could render the virus resistant to amantadine (*11*,*12*).

Our epidemiologic investigation revealed that the farm of the affected flock had multiple biosecurity deficiencies. For example, the farm was 80 meters from a road on which a high volume of garbage trucks traveled. Additionally, the farm had neither procedures for control and disinfection of vehicles and pedestrians entering the property, nor facilities to exclude rodents and wild birds, and many sparrows shared the feed with the egg-laying chickens in the poultry house.

## Conclusions

HPAI mass vaccination played a crucial role in HPAI control in China. However, this study demonstrated multiple disadvantages of HPAI mass vaccination, which had been suspected (*13*,*14*). For example, this study showed that H5N1 subtype HPAI virus has evolved into multiple H5N2 genotypes, which are all likely vaccine-escape variants, suggesting that this virus can easily evolve into vaccine-escape variants. This observation suggests that HPAI mass vaccination, which is highly effective in the beginning of an outbreak, may lose its effectiveness with time unless the vaccine strains are updated. Moreover, this study showed that vaccinated chicken flocks can be infected with vaccine-escape variants without signs of illness. Thus HPAI mass vaccination may increase shedding of the virus by infected chickens that otherwise would likely exhibit signs of illness and die soon after infection; therefore, HPAI mass vaccination may increase spread of a virus that otherwise would be easily identified by observation of clinical signs. 

Currently, HPAI mass vaccination in poultry should not be stopped; otherwise, many HPAI outbreaks could likely occur in poultry farms with limited biosecurity. Conversely, HPAI mass vaccination in China cannot be expected to have a progressive effect because the practice leads to silent spread of vaccine-escape variants selected in the host immunologicpressure induced against vaccine strains. We propose that the only way out of this dilemma is to strengthen the strategy published previously, which covers the following components: education, biosecurity, rapid diagnostics and surveillance, and elimination of infected poultry (*14*). Mass vaccination should be used as an additional tool within this 4-component strategy, not in place of the 4 components.

Technical Appendix Phylogenetic relationships of some avian influenza A(H5N2) highly pathogenic viruses, and other avian influenza viruses circulating during recent years.
