# Supplementary material for: Subclinical Highly Pathogenic Avian Influenza Virus Infection among Vaccinated Chickens, China
Source: Emerg Infect Dis. 2014 Dec;20(12):2152–4. doi: 10.3201/eid2012.140733 (PMC4257838; doi:10.3201/eid2012.140733)
Supplement: Technical Appendix — Phylogenetic relationships of some avian influenza A(H5N2) highly pathogenic viruses, and other avian influenza viruses circulating during recent years. [file 14-0733-Techapp-s1.pdf]

DOI: http://dx.doi.org/10.3201/eid2012.140733

Subclinical Highly Pathogenic Avian Influenza Virus Infection among Vaccinated Chickens, China

Technical Appendix Figures 1–7 show phylogenetic relationships of some H5N2 subtype highly pathogenic avian influenza viruses (marked with triangles) and other avian influenza viruses circulating during recent years based on the nucleotide sequences of their polymerase basic 2 protein (Technical Appendix Figure 1), polymerase basic 1 protein (Technical Appendix Figure 2), polymerase acidic (Technical Appendix Figure 3), NP (Technical Appendix Figure 4), NA (Technical Appendix Figure 5), M (Technical Appendix Figure 6), and NS (Technical Appendix Figure 7) genes, respectively. The viruses with the same branch color are assumed to be in the same lineage; lineages with red branches (lineage A) are assumed to be derived from H9N2 subtype avian influenza viruses, and the lineages with green, blue or pink branches cover at least one H5N2 subtype highly pathogenic avian influenza virus isolated in China in recent years. Bootstrap values are provided at relevant nodes. PB, polymerase basic, PA, polymerase acidic; NP, nucleoplasmic protein; NA, neuraminidase; M, matrix protein; NS, nonstructural protein.

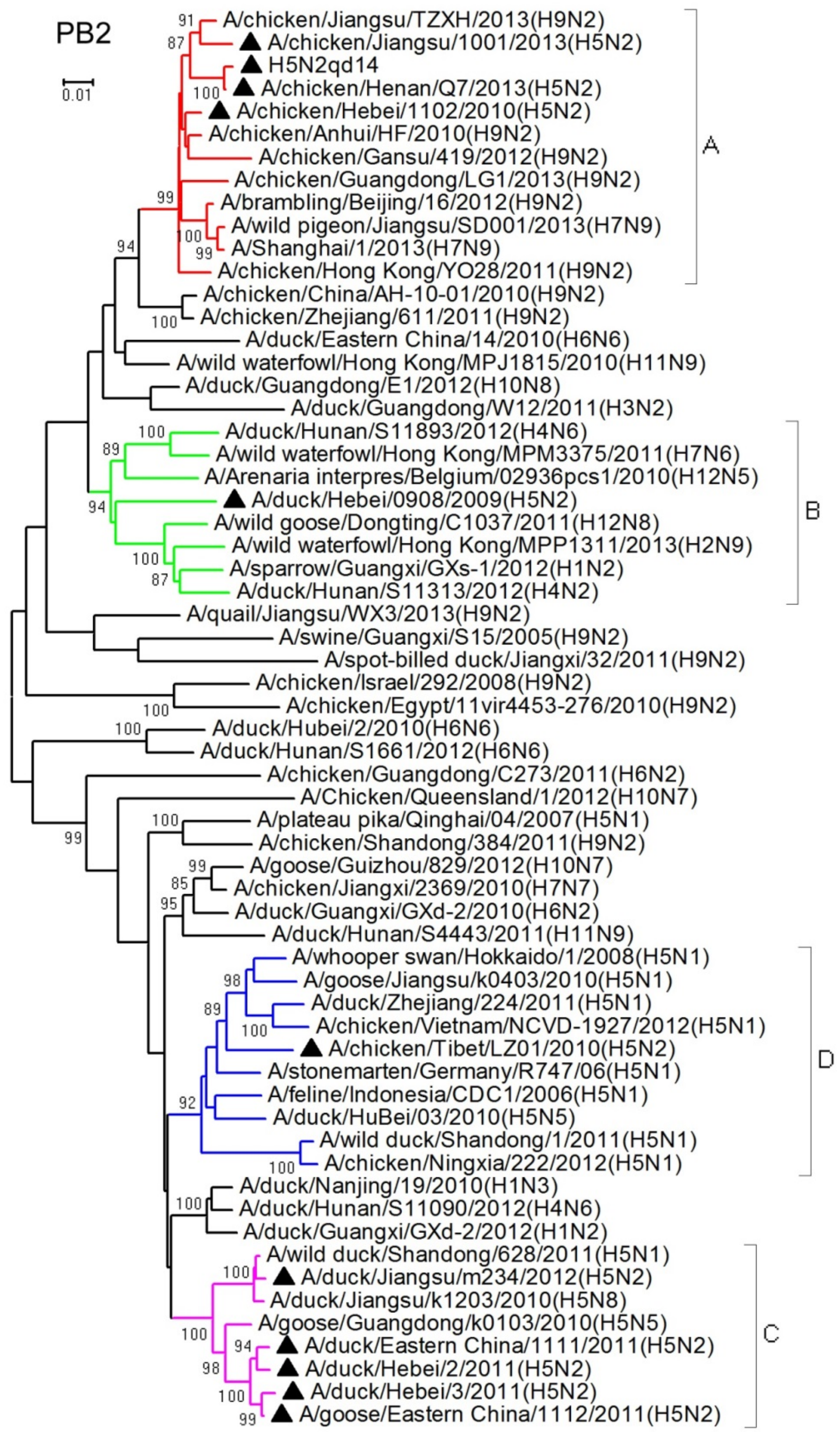

Technical Appendix Figure 1.

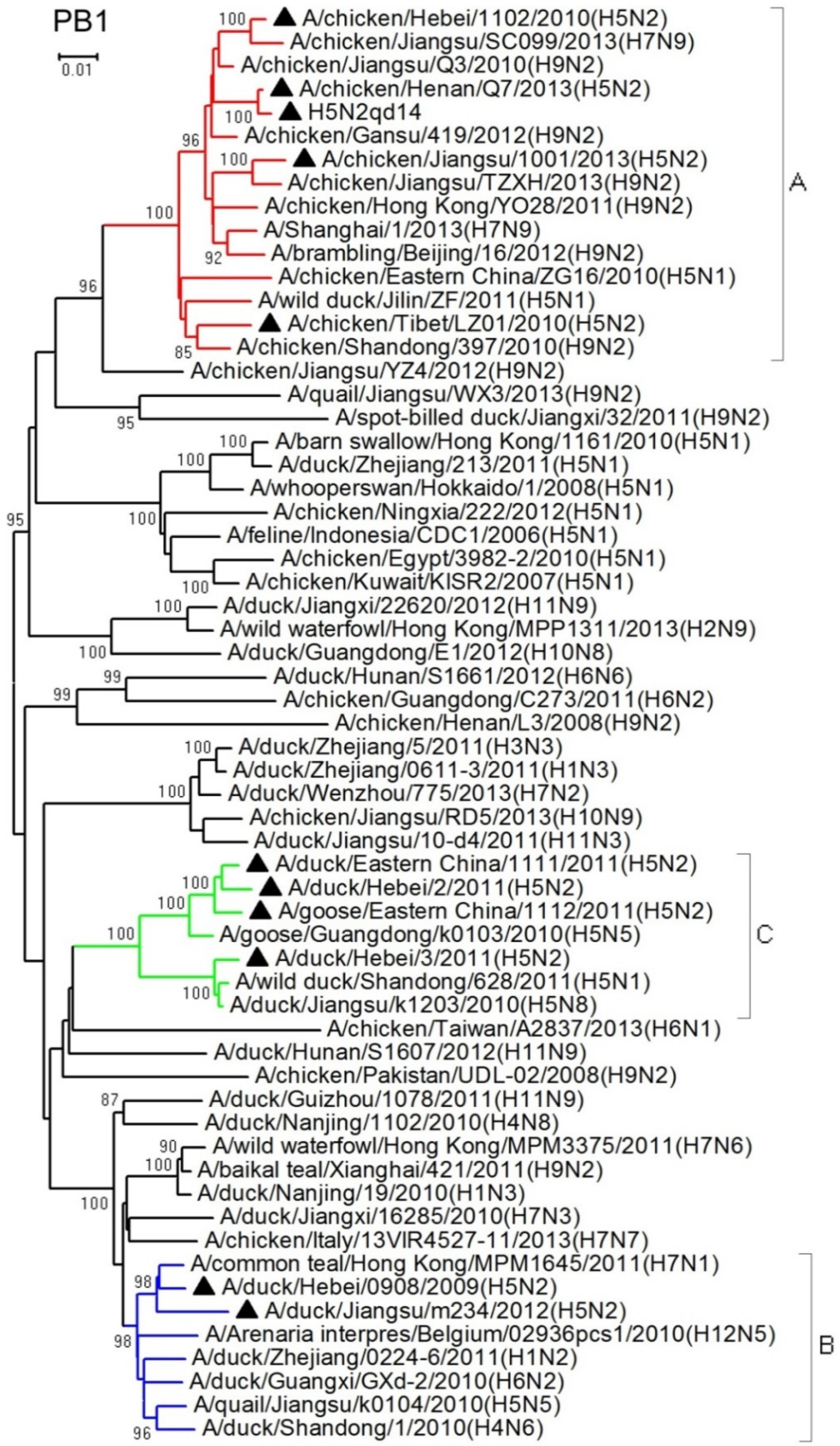

Technical Appendix Figure 2.

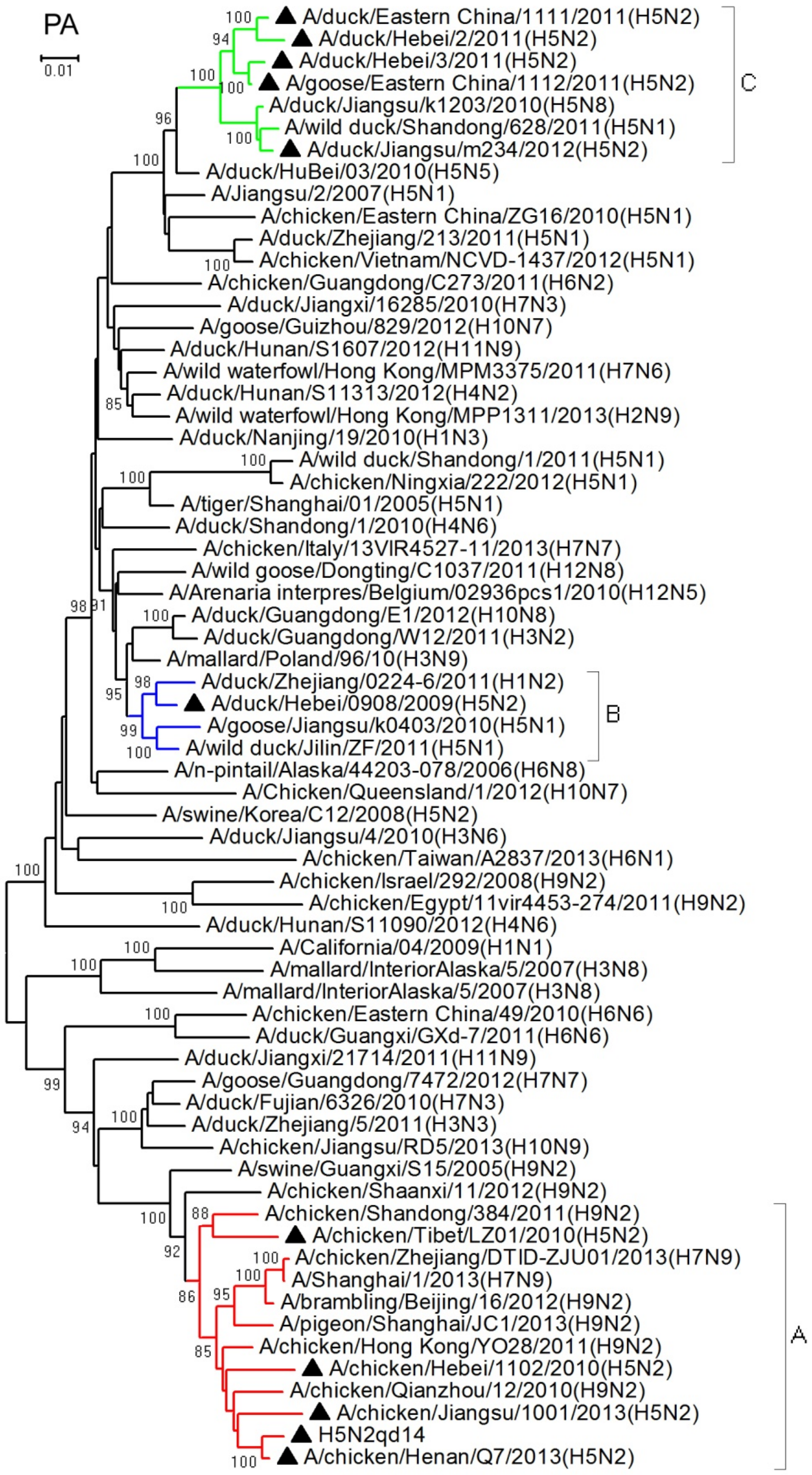

Technical Appendix Figure 3. Scale bar represents polymerase acidic units per site.

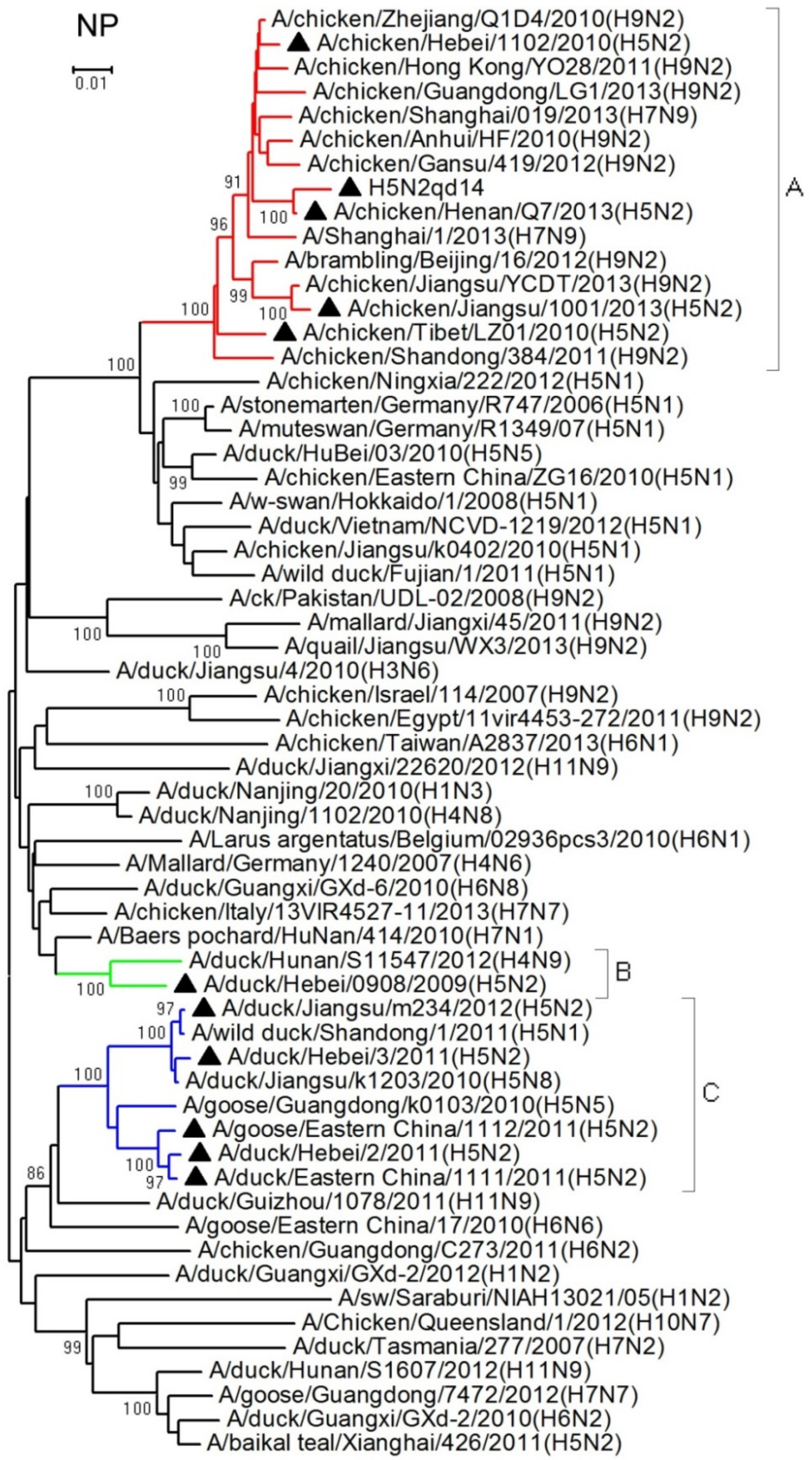

Technical Appendix Figure 4

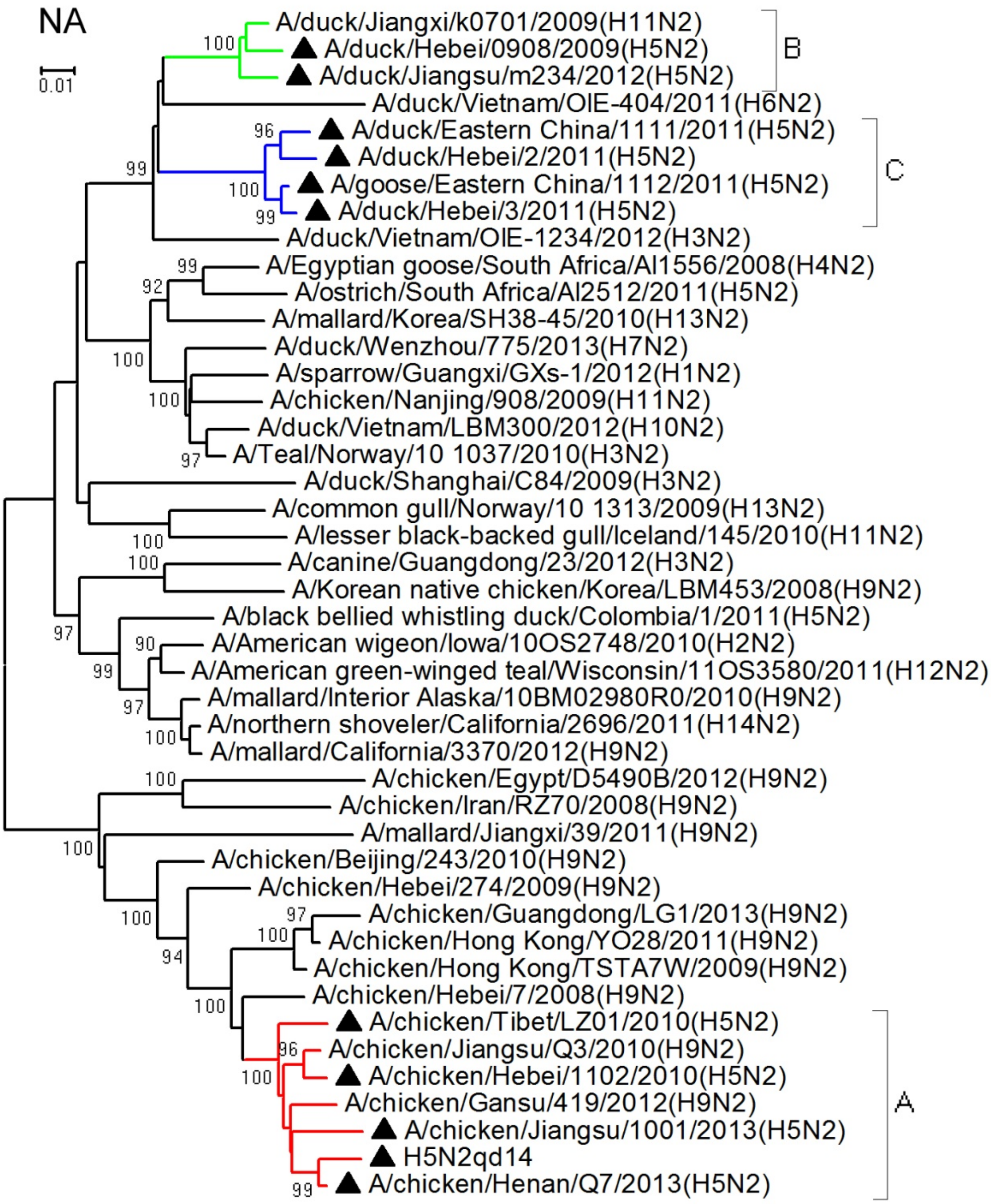

Technical Appendix 5

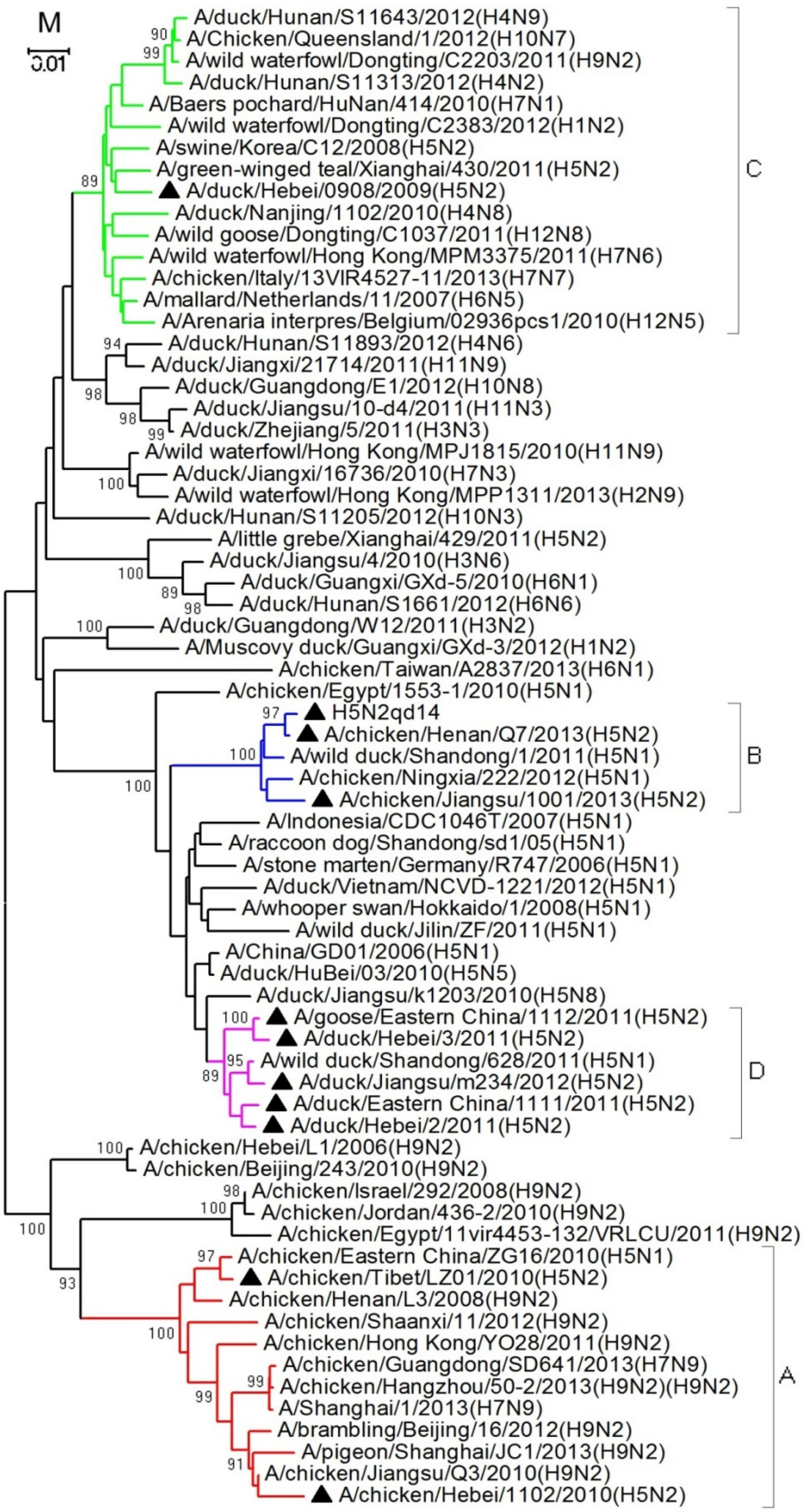

Technical Appendix Figure 6

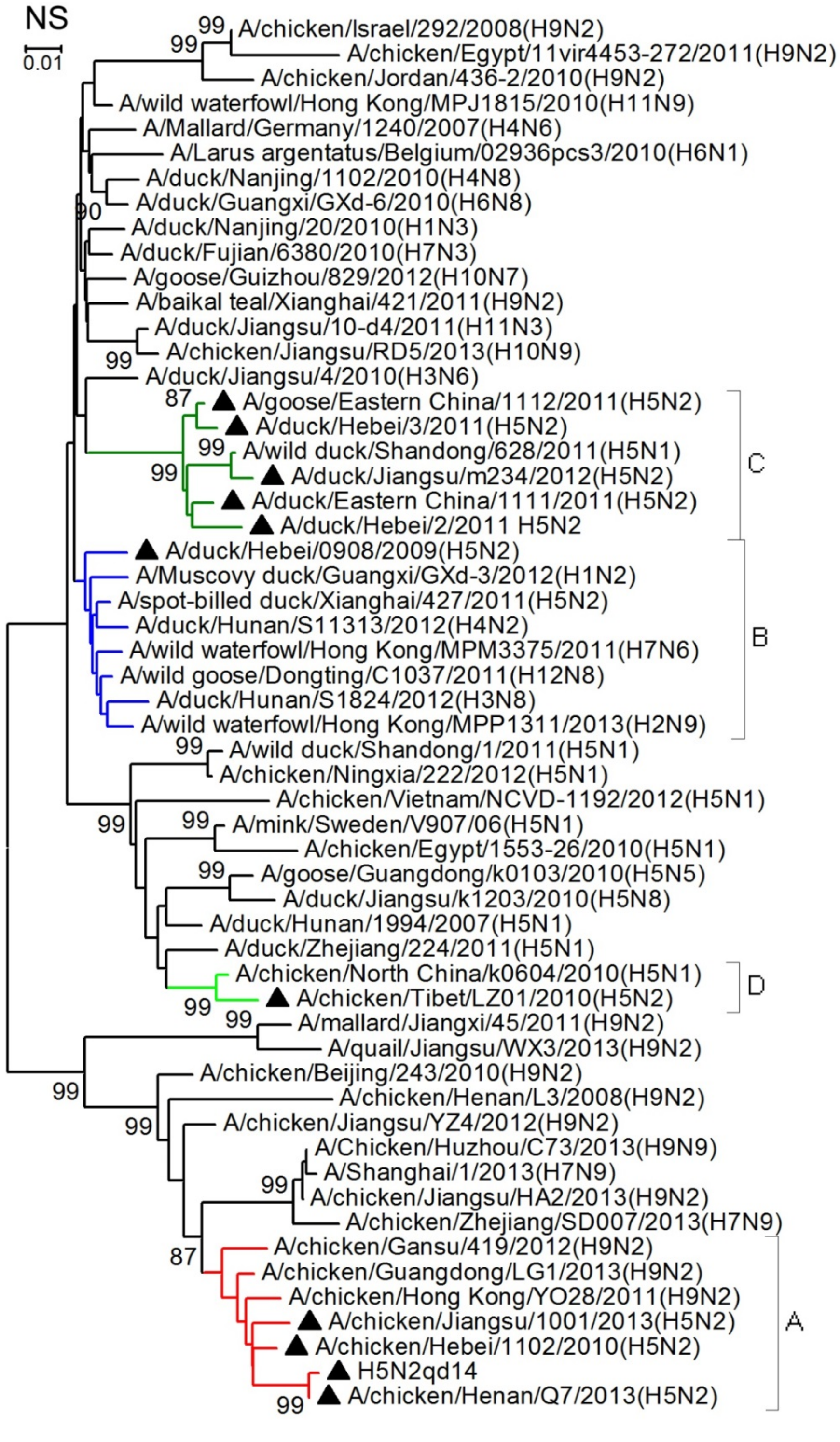

Technical Appendix Figure 7
